# Mitochondrial dysfunction and programmed cell death in Alzheimer’s disease: A retrospective bioinformatics study

**DOI:** 10.1097/MD.0000000000048105

**Published:** 2026-03-20

**Authors:** Hua-xiong Zhang, Hong-yan Li

**Affiliations:** a Department of Neurology, Xinjiang Uygur Autonomous Region People’s Hospital, Urumqi, China; b Xinjiang Clinical Research Center for Stroke and Neurological Rare Diseases, Urumqi, China.

**Keywords:** Alzheimer’s disease, bioinformatics analysis, key genes, mitochondria, programmed cell death

## Abstract

Alzheimer’s disease (AD) is a major cause of dementia, and this paper explores the unclear roles of mitochondrial dysfunction and programmed cell death in AD. Differentially expressed genes (DEGs) were identified using AD datasets GSE63061 and GSE63060 from the Gene Expression Omnibus. DEGs were intersected with mitochondria-related genes and programmed cell death-related genes to obtain DEGs (in AD) intersected with mitochondrial-related genes and DEGs (in AD) intersected with programmed cell death-related genes. Correlation analysis of these DEGs was used to identify candidate genes. Machine learning algorithms were applied to key genes, followed by functional enrichment, network construction, immune infiltration analysis, drug prediction, and expression validation. Two key genes, superoxide dismutase 1 (SOD1) and translocase of the outer mitochondrial membrane 7 (TOMM7), were identified and linked to pathways like ribosome and chemokine signaling. A strong positive correlation (0.76, *P* < .001) was found between them. Immune analysis showed differences in 11 immune cells between AD and controls, with TOMM7 positively linked to activated CD8 T cells and negatively to myeloid-derived suppressor cells. SOD1 and TOMM7 are regulated by 4 miRNAs and 71 long noncoding RNAs (lncRNAs). Seventeen potential AD drugs, including urea and nitric oxide, were predicted. Two key genes, SOD1 and TOMM7, related to mitochondria and PCD, were identified as potential targets for understanding AD’s etiology, detection, and therapeutic approaches.

## Graphical abstract

**Figure:**
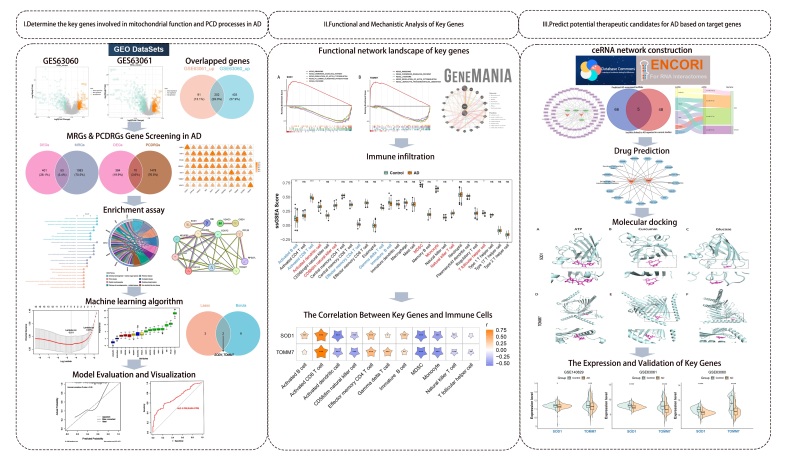

## 1. Introduction

Alzheimer’s disease (AD) is the predominant type of dementia among elder adults.^[1]^ As age serves as the key risk factor for AD, the rising life expectancy will consequently lead to a rapid increase in the prevalence of AD patients.^[2]^ By 2050, dementia rates are expected to double in Europe and triple globally, with even higher estimates using a biological definition of AD.^[3]^ AD is a neurodegenerative disorder distinguished by neuropathological changes like amyloid-β plaques and neurofibrillary tangles (NFTs), causing a progressive worsening of cognitive function, with the rate of decline and specific impairments varying among individuals. As the disease advances, neuronal damage spreads throughout the brain, necessitating increased support from caregivers to assist with daily tasks and ensure the individual’s safety. Additionally, individuals with AD may exhibit alterations in mood, personality, and behavior.^[4,5]^ Ultimately, AD is a fatal brain disease. Research suggests that individuals aged 65 and above typically survive for 4 to 8 years following AD diagnosis, although some may live up to 2 decades.^[2,6-14]^ Because of this, AD has risen to the status of one of the 21st century’s most expensive, deadly, and burdensome disorders.^[3]^ Unfortunately, the etiology of AD remains incompletely elucidated, and current treatment options are lacking in efficacy. Thus, it is essential to find innovative biomarkers characterized by heightened sensitivity and specificity for the purpose of diagnosing and targeting therapeutic interventions for AD. Such biomarkers will provide a foundation for clinical investigations and serve as a point of reference.

Mitochondria, the primary energy-producing system in eukaryotic cells that produces adenosine triphosphate (ATP) through oxidative phosphorylation, are versatile organelles involved in multiple biological processes like metabolism, cell homeostasis, and signaling.^[15]^ There has been plenty of research showing that mitochondrial dysfunction is important in AD.^[16-19]^ As per previous reports, AD is linked to mitochondrial dysfunction, such as excessive ROS production, decreased ATP generation, and disrupted calcium homeostasis. This leads to damage to mitochondria, synaptic dysfunction, and, ultimately, AD progression.^[20]^ Aβ plaques and NFTs also impair mitochondrial function, worsening neuronal deficits and contributing to neurodegeneration in AD.^[21]^ Previous research indicates that removing the damaged mitochondria, called mitophagy, is diminished in AD. This leads to a buildup of dysfunctional mitochondria, which contributes to cognitive decline and synaptic dysfunction by promoting the accumulation of Aβ and Tau proteins.^[22]^ Meanwhile, boosting mitophagy eliminates excessive tau phosphorylation in human neurons, improves memory in transgenic animals, and could be a promising treatment for AD.^[23]^ Last but not least, Mitochondria are central to various cell death pathways, influencing both apoptotic and nonapoptotic programmed cell death (PCD). Dysfunction in these pathways can lead to age-associated disorders like neurodegenerative, cardiovascular, and metabolic disorders.^[24]^

PCD refers to the controlled process by which cells undergo regulated death. This phenomenon encompasses a variety of cell death mechanisms that are initiated through intrinsic programming, which occurs during organismal growth and development, as well as in response to environmental and pathological stimuli.^[25]^ PCD is also a significant contributor to the onset of degenerative diseases,^[26]^ especially AD. In terms of pathology, abnormal cell death stimulation is a prevalent feature in AD pathogenesis, causing neuron loss and cellular dysfunction.^[27]^ Meanwhile, a previous study found that TUNEL staining showed higher DNA fragmentation in AD-affected brain tissue than in controls, indicating that neurodegeneration in AD patients may result from apoptosis.^[28]^ Additionally, research has shown that activated brain immune cells can induce PCD, leading to the secretion of proinflammatory cytokines, enhancing the innate immune response, and aiding in the removal of Aβ plaques and aggregated Tau proteins.^[29-31]^ Nevertheless, chronic neuroinflammation resulting from apoptosis can exacerbate AD.^[32,33]^ An interaction of these factors may cause a vicious cycle of deterioration in AD.

So, new insights into AD clinical trials have been provided by medicines based on mitochondria-associated PCDs, which demonstrate promising therapeutic potential. Nevertheless, there is a lack of clear characterization of the mechanisms at play and the specific relationships between mitochondrial function, AD, and PCD. This study utilized bioinformatics analysis to detect crucial genes associated with mitochondria and PCD in AD. Functional networks of these key genes were constructed, and potential target drugs were predicted to detect potential therapeutic options for AD. This investigation provides an innovative scientific foundation for understanding AD pathogenesis and treatment.

## 2. Materials and methods

All gene expression data used in this study were obtained from the publicly available Gene Expression Omnibus (GEO) database (https://www.ncbi.nlm.nih.gov/gds). These datasets (GSE63061, GSE63060, and GSE140829) contain de-identified, anonymized human peripheral blood transcriptomic data. As this investigation is a retrospective bioinformatics analysis of publicly available, non-identifiable data, it was exempt from ethical review and approval by an institutional review board. Furthermore, informed consent from individual participants was not required, as the data cannot be linked back to any specific individual.

### 2.1. Data collection

**Figure 1.**
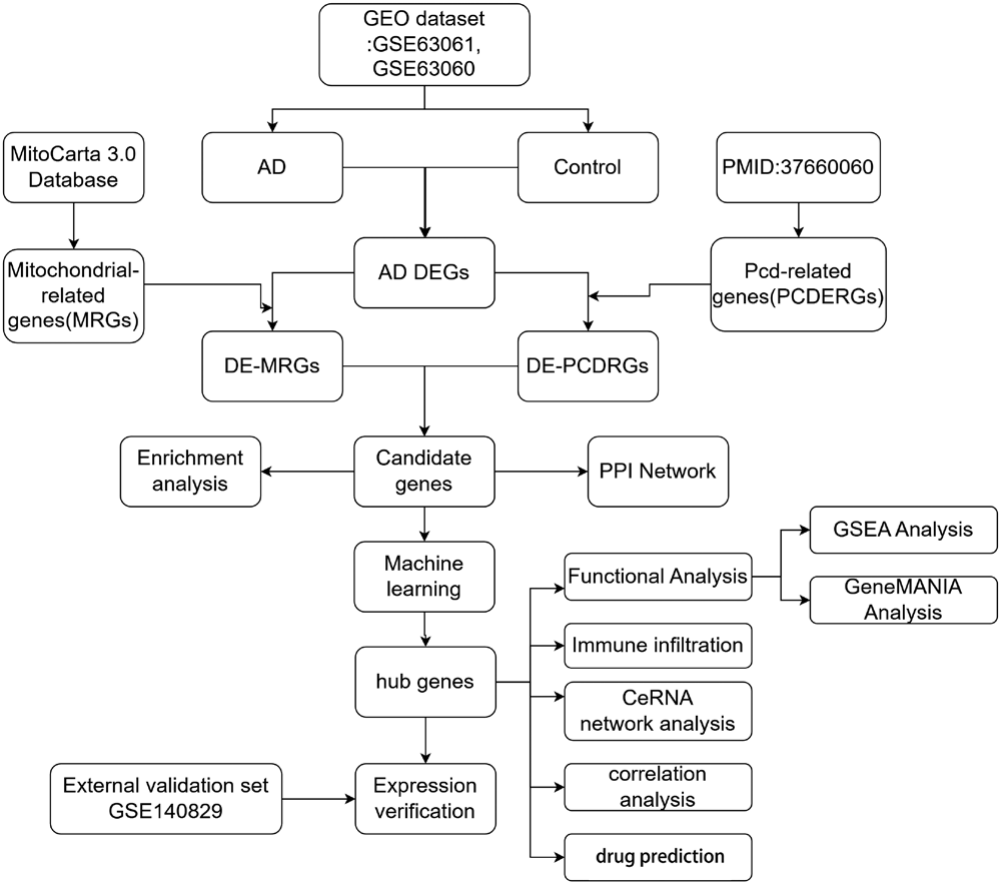
Flowchart. AD = Alzheimer’s disease, DEG = differentially expressed gene, GEO = Gene Expression Omnibus, GSEA = gene set enrichment analysis, MRG = mitochondria-related gene, PPI = protein–protein interaction.

This retrospective bioinformatics study utilized gene expression data from the GEO database (https://www.ncbi.nlm.nih.gov/gds) were employed in this investigation. Specifically, GSE63061, comprising 388 blood samples and 273 samples identified as Control (134) and AD (139), sequenced on the GPL10558 platform, serves as the training set. GSE63060, containing 329 blood samples with 104 Control and 145 AD samples, sequenced on the GPL6947 platform, served as an auxiliary training set. The GSE140829 dataset, with 587 blood samples, including 249 Control and 204 AD samples, sequenced on the GPL15988 platform, was used for expression validation. The 1136 mitochondria-related genes (MRGs) were derived from the reference article,^[34]^ while programmed cell death-related genes (PCDRGs) were compiled from 18 PCD modes and key regulatory genes identified in a study,^[35]^ totaling 1548 PCDRGs. A detailed flow chart of the study process is presented in Figure 1.

### 2.2. Differential expression analysis

In the GSE63061 dataset and the GSE63060 dataset, differential expression analysis was performed via the limma package (v 1.36.0)^[36]^ to obtain DEGs1 and DEGs2. Visualizations of the differentially expressed genes (DEGs; |log_2_FC| > 0.1 and *P*.adjust < .05), including volcano plots and heat maps, were created with the ggplot2 (v 3.3.6)^[37]^ and the ComplexHeatmap (v 2.12.1) packages.^[38]^ The intersection of DEGs1 and DEGs2, categorized by their upregulated and downregulated status, was performed to derive the set of DEGs and visualized by the ggvenn package (v 0.1.9).^[39]^

### 2.3. Identification and functional annotation of candidate genes

Differentially expressed genes (in AD) intersected with mitochondrial-related genes (DE-MRGs) and differentially expressed genes (in AD) intersected with programmed cell death-related genes (DE-PCDRGs) were created by intersecting DEGs with MRGs and PCDRGs, respectively. The ggvenn was used for visualization.^[39]^ The Spearman correlation analysis of DE-MRGs and DE-PCDRGs was performed by the psych package (v 2.2.9)^[40]^ (|cor| > 0.9 and *P*value < .05). The correlation heatmap was visualized via the ggcor package (v 0.9.8.1).^[41]^ The final filtered DE-MRGs and DE-PCDRGs were merged into 1 set, defined as candidate genes, and the resulting gene pairs were visualized by heatmap using ggcor.^[41]^

To identify prevalent roles and related pathways, candidate genes underwent enrichment analysis for Gene Ontology (GO) and Kyoto Encyclopedia of Genes and Genomes (KEGG), utilizing the clusterProfiler package (v 4.7.1.001)^[42-45]^ (*P* < .05). The outcomes were visualized by the ggpubr package (v 0.5.0) and the GOplot package (v 1.0.2).^[46]^ Furthermore, the STRING database (http://string.embl.de/) was employed to build a protein–protein interaction network, aiming to explore whether there were interactive relationships among the candidate genes (low confidence > 0.4).

### 2.4. Identification of key genes

For further screening of key genes, the gene intersection acquired from Least Absolute Shrinkage and Selection Operator regression analysis employing the glmnet package (v 4.1-6)^[47]^ and genes identified by the Boruta algorithm via the Boruta package (v 8.0.0)^[48]^ was calculated via the ggvenn.^[39]^ The genes resulting from this intersection were defined as key genes.

Verifying the expression trends of key genes was conducted by visualization and the Wilcoxon rank sum test in the training set GSE63061 and auxiliary training set GSE63060 and further confirmed in the external validation set GSE140829.

### 2.5. A nomogram construction

To evaluate the diagnostic value of key genes, a nomogram model was created via the rms package (v 6.3-0)^[49]^ within the GSE63061. The model’s prognostic precision was assessed by the analysis of calibration plots and receiver operating characteristic (ROC) curves. A Spearman correlation analysis of the 2 key genes was carried out via the psych,^[40]^ and the correlation heatmap was visualized via the ggplot2.^[37]^

### 2.6. Gene functional network construction

To further explore some relevant functions in key genes, Spearman correlation analysis was conducted via the psych.^[40]^ The clusterProfiler^[50]^ was utilized to conduct pathway enrichment analysis through gene set enrichment analysis (GSEA; |NES| > 1, *P*.adjust < .05). The background gene set file, c2.cp.kegg.v7.0.symbols.gmt, was acquired from the GSEA portal (http://www.gsea-MSigdb.org/gsea/msigdb). The key genes were analyzed and networks were constructed via the GeneMANIA online tool (http://genemania.org/).

### 2.7. Immune infiltration analysis

The ssGSEA algorithm from the GSVA package (v 1.44.5)^[51]^ was utilized to determine immune cell scores for every sample, and stacked bar graphs representing these scores were created for the control and AD groups using the ggplot2.^[37]^ The box plots of 28 immune cell scores were plotted, and a Wilcoxon rank test was performed between the control and AD groups in the training set GSE63061. Spearman’s correlations between key genes and differential immune cells were analyzed, with plots created via the ggcor.^[41]^

### 2.8. Regulatory network construction and drug prediction

To further elucidate the interactions between different RNA molecules, predictions of miRNAs associated with key genes were made via the miRTarbase database (https://mirtarbase.cuhk.edu.cn/~miRTarBase/miRTarBase_2025/php/index.php). Based on the obtained miRNAs, lncRNAs (clipExpNum > 5) were predicted in the starBase database to create competing endogenous RNA (ceRNA) networks. Then, we identified key AD-related lncRNAs by intersecting those reported in the current literature^[52-61]^ with our predicted lncRNAs. We utilized the R “Sankeywheel” package to create a Sankey diagram visualizing the key LncRNA-miRNA-mRNA relationships. The coremine database (https://www.coremine.com/medical/#search) was utilized to predict the target drugs of key genes, and the outcomes were depicted using Cytoscape (v 3.8.2).^[62]^ Protein structures for key genes were sourced from the PDB database, and compound structures from the PubChem database (https://www.rcsb.org/). The protein structures were molecularly docked with the corresponding compounds using Autodock software (v 1.1.2),^[63]^ the binding energies between the ligands and receptors were calculated, and visualization of the outcomes was achieved with PyMOL (v 2.5.2)^[64]^ (molecular binding energy ≤ –5.0 kcal/mol).

### 2.9. Statistical analysis

Data analysis was conducted via R software (v 4.1.0, Urumqi, Xinjiang Uygur Autonomous Region, China), with the Wilcoxon test employed to evaluate group variations (*P* < .05).

## 3. Results

### 3.1. Acquisition of the 454 DEGs

**Figure 2.**
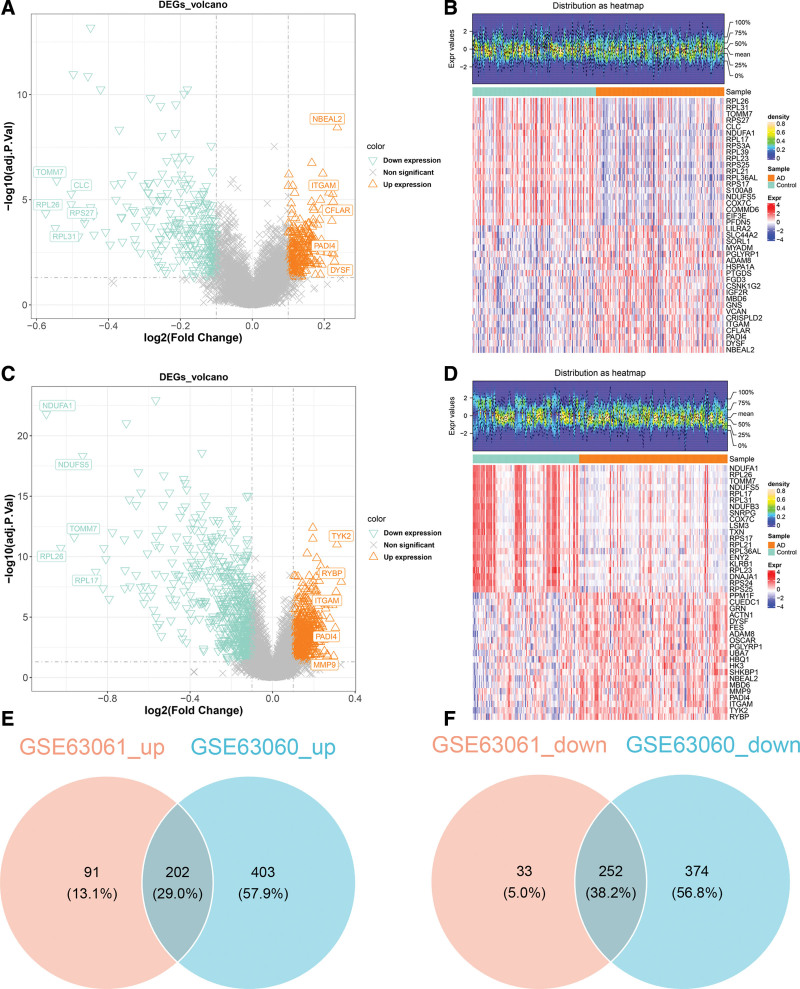
Integration and differential expression analysis of the AD dataset. (A) Volcano plot of DEGs in GSE63061 dataset (orange triangles indicate up-regulated genes, green inverted triangles denote down-regulated genes, and gray X’s mark nonsignificant genes). (B) Heatmap of the top 40 upregulated and downregulated DEGs in GSE63061 dataset (the top graph illustrates the density distribution of differential gene expression. In the bottom graph, the *x*-axis denotes the sample, and the *y*-axis denotes the gene. Green indicates the control sample, while orange–red represents the AD sample. Red signifies highly expressed genes, and blue indicates poorly expressed genes). (C) Volcano plot of DEGs in GSE63060 dataset. (D) Heatmap of the top 40 upregulated and downregulated DEGs in GSE63060 dataset. (E) The overlap of genes that are upregulated in both the GSE63061 and GSE63060 datasets. (F) The overlap of genes that are downregulated in both the GSE63061 and GSE63060 datasets. AD = Alzheimer’s disease, DEG = differentially expressed gene.

A total of 578 DEGs1, with 293 upregulated and 285 downregulated, were screened in the GSE63061 dataset (Fig. 2A, B). The 1231 DEGs2 were screened in the GSE63060 dataset, including 605 upregulated and 626 downregulated (Fig. 2C, D). Taking the intersection of DEGs1 and DEGs2, 454 DEGs were obtained, including 202 upregulated and 252 downregulated (Fig. 2E, F).

### 3.2. Enrichment analysis and identification of candidate genes

**Figure 3.**
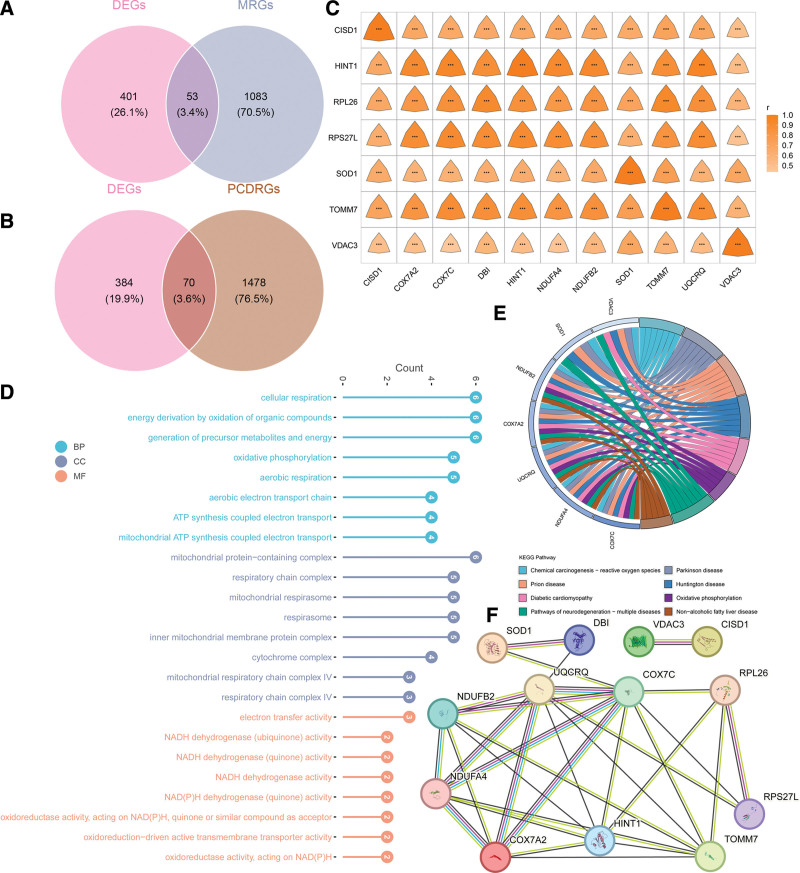
Candidate gene enrichment analysis and PPI network construction. (A) The intersection gene of AD DEGs and MRGs. (B) The intersection gene of AD DEGs and PCDRGs. (C) Correlation heat map of DE-MRGs and DE-PCDRGs (the horizontal axis shows DE-PCDRGs, while the vertical axis displays DE-MRGs. Orange indicates a positive correlation, with significance marked as ***, *P* < .001). (D) GO enrichment analysis of candidate genes. (E) KEGG enrichment analysis of candidate genes. (The circle’s left half shows genes with colors indicating logFC values: blue for down-regulation and darker shades for larger differences.) The right half displays KEGG pathways, each in a distinct color). (F) The PPI network of candidate genes. AD = Alzheimer’s disease, DEG = differentially expressed gene, DE-MRGs = differentially expressed genes (in AD) intersected with mitochondrial-related genes, DE-PCDRGs = differentially expressed genes (in AD) intersected with programmed cell death-related genes, GO = Gene Ontology, KEGG = Kyoto Encyclopedia of Genes and Genomes, MRG = mitochondria-related gene, PCDRG = programmed cell death-related gene, PPI = protein–protein interaction.

The intersection of DEGs was conducted with MRGs to acquire 53 DE-MRGs and 70 DE-PCDRGs were obtained by overlapping DEGs and PCDRGs (Fig. 3A, B). Spearman’s correlation analysis was performed between DE-MRGs and DE-PCDRGs, and a total of 11 DE-MRGs and 7 DE-PCDRGs were screened. The concatenation of the 2 resulted in 13 genes (CISD1, HINT1, superoxide dismutase 1 (SOD1), translocase of the outer mitochondrial membrane 7 (TOMM7), and VDAC3 genes belonged to both MRGs and PCDRGs), which were defined as candidate genes (Fig. 3C).

To understand the molecular function and mechanism, we analyzed the candidate genes for GO and KEGG enrichment. They were mainly enriched in GO enrichment analysis in cellular respiration, intrinsic apoptotic signaling pathway, mitochondrial inner membrane, and other functional entries (Fig. 3D). They were mainly enriched in KEGG enrichment analysis in the following pathways: Chemical carcinogenesis - ROS, AD, and Parkinson’s disease (Fig. 3E). Through the protein–protein interaction network construction, the interaction network of 13 genes was acquired, which had a total of 13 nodes and 31 edges. These included the following relationship pairs: SOD1-DBI and TOMM7-COX7C (Fig. 3F).

### 3.3. SOD1 and TOMM7 were identified as key genes

**Figure 4.**
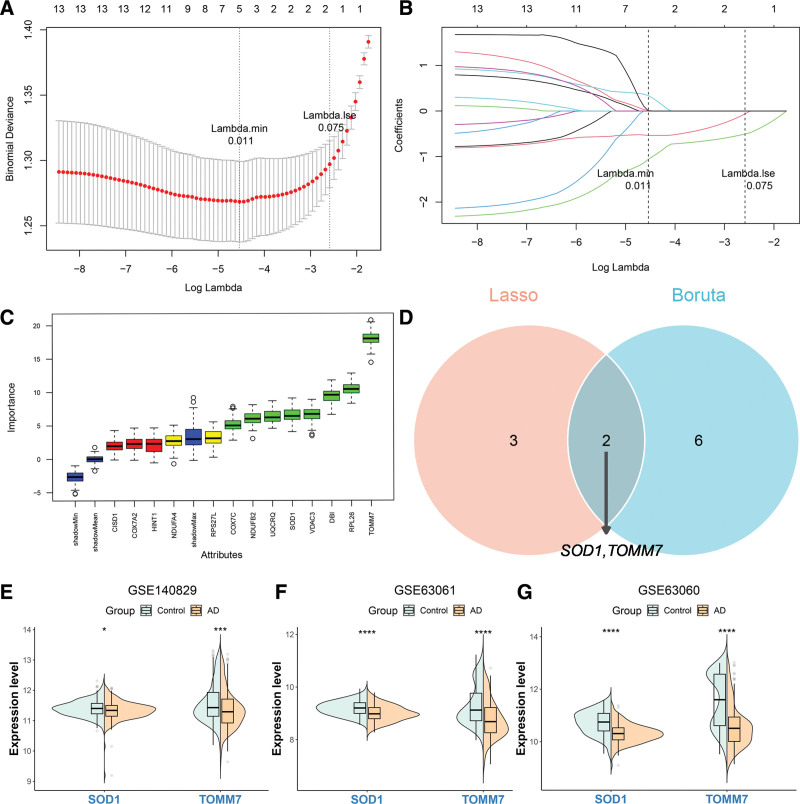
Identification of potential diagnostic biomarkers associated with mitochondria and PCD in AD using machine learning. (A, B) Identification of the minimum value and lambda value for diagnostic biomarker selection using the LASSO logistic regression algorithm. (C) The Boruta machine learning algorithm is employed to identify and evaluate candidate genes (In the figure, green shows important features, yellow indicates unclear ones, and blue boxes display the minimum, average, and maximum *Z*-values of the shaded attributes). (D) Venn diagram representing the shared genes between 2 unique machine learning strategies. (E–G) Expression verification of key genes. AD = Alzheimer’s disease, LASSO = Least Absolute Shrinkage and Selection Operator, PCD = programmed cell death.

Least Absolute Shrinkage and Selection Operator regression analysis of 13 candidate genes yielded 5 genes: COX7A2, HINT1, SOD1, TOMM7, and Ribosomal Protein S27-like (RPS27L; Fig. 4A, B). Thirteen candidate genes were analyzed via the Boruta algorithm. Eight genes were screened, namely COX7C, DBI, NDUFB2, SOD1, TOMM7, UQCRQ, VDAC3 and RPL26 (Fig. 4C). By intersecting the gene sets identified through screening with 2 machine learning algorithms, SOD1 and TOMM7 were found to be common to both MRGs and PCDRGs. These 2 genes were, therefore, selected as key candidates (Fig. 4D). Finally, validation of key gene expression confirmed that the trends observed in GSE140829 were consistent yet significantly divergent from those in GSE63061 and GSE63060 (Fig. 4E–G).

### 3.4. Strong positive correlation between SOD1 and TOMM7

**Figure 5.**
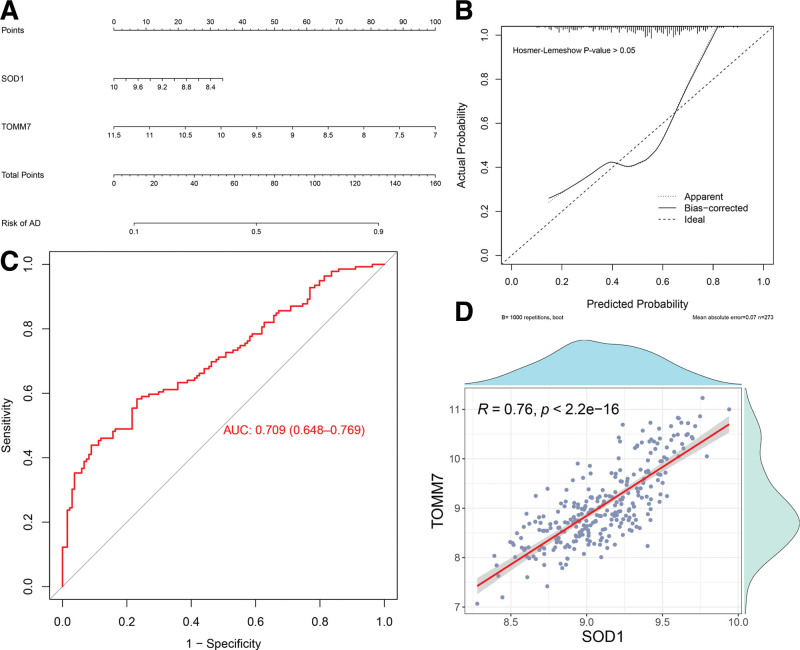
Nomogram model construction and evaluation. (A) Nomogram model. (B) Nomogram model calibration curve. (C) ROC curve of Nomogram model. (D) Key genes correlation fitting curve. ROC = receiver operating characteristic.

A predictive nomogram model was created to estimate the likelihood of AD diagnosis depending on the expression levels of SOD1 and TOMM7 in GSE63061. Through regression analysis, we obtained the predictive equation of the model: *y* = 13.46 + (–0.68) × SOD1 expression level + (–0.80) × TOMM7 expression level (Fig. 5A). The calibration curves based on the nomogram model showed good diagnostic results in this model for AD samples (Fig. 5B). Similarly, the area under the ROC curve (AUC) was 0.709, reaffirming high predictive accuracy (Fig. 5C). Meanwhile, Spearman’s analysis showed a robust positive correlation (0.76) between SOD1 and TOMM7 (*P* < .001; Fig. 5D).

### 3.5. Functional network landscape of key genes

**Figure 6.**
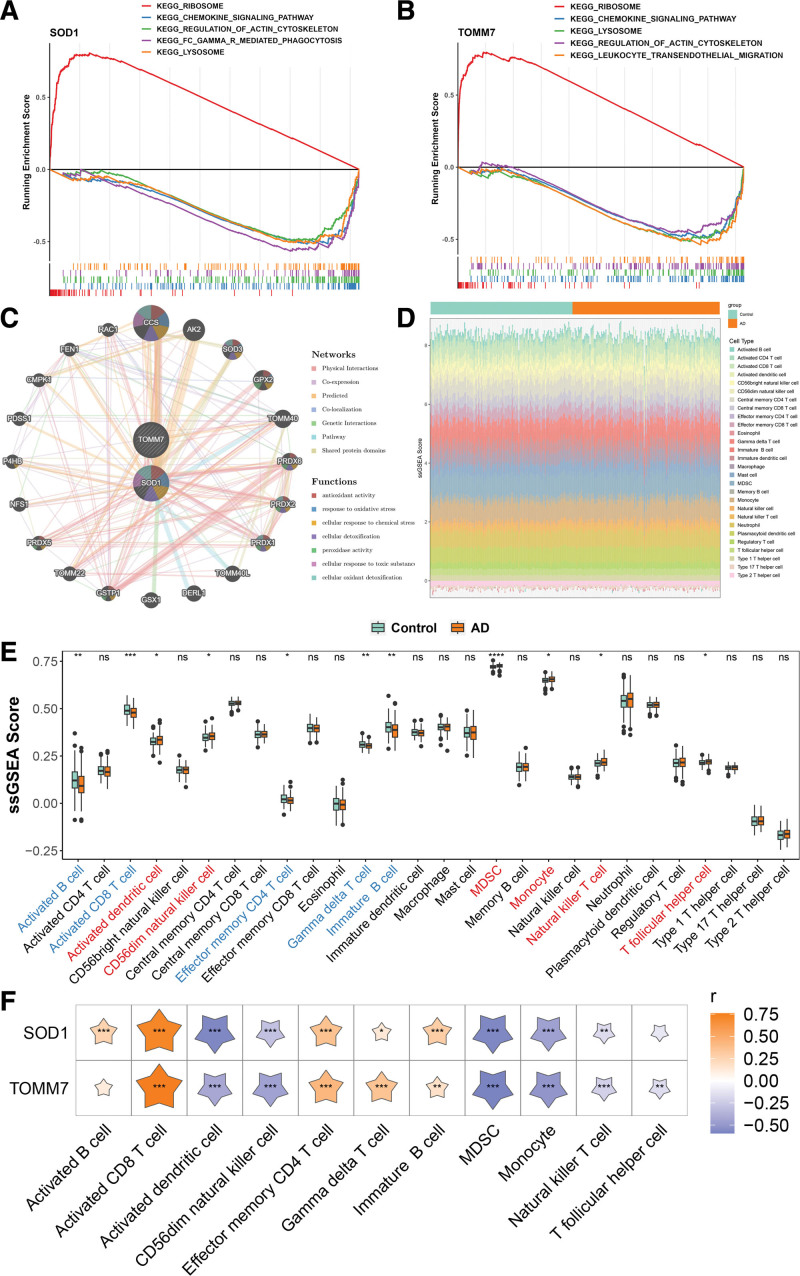
A comprehensive analytical diagram integrating GSEA, Genemia genes, and immune infiltration. (A, B) GSEA analysis of key genes. (C) GeneMANIA analysis of key genes. (D) Bar graph of immune cell infiltration score accumulation. (E) Box plots of immune cell infiltration scores in the Control and AD groups. (F) Heat map of key genes associated with immune cells (the *x*-axis shows immune cells, the y-axis shows key genes, and the pentagram indicates positive or negative correlations. Darker colors signify stronger correlations. Significance levels: ns, *P* > .05, *P < .05, **P < .01, ***P < .001). AD = Alzheimer’s disease, GSEA = gene set enrichment analysis.

Single-gene GSEA pathway enrichment analysis of the key genes illustrated that they were mainly enriched in the ribosome, chemokine signaling pathways, regulation of actin cytoskeleton, and lysosome (Fig. 6A, B). Utilizing the GeneMANIA database, a network was established for the 2 key genes, revealing 20 genes with significant interactions. The related functions were cellular detoxification, antioxidant activity, and response to oxidative stress (Fig. 6C).

### 3.6. Immune infiltration mapping of key genes

Analysis of immune cell scores across samples revealed 11 immune cell infiltration scores that illustrated significant variations between the control and AD groups. Such as monocytes, T follicular helper, activated B, activated dendritic, CD56dim natural killer, effector memory CD4 T, gamma delta T, immature B, activated CD8 T, natural killer T, and myeloid-derived suppressor cells (MDSC). Activated B, effector memory CD4 T, activated CD8 T, gamma delta T, and immature B cells were less abundant, and the remaining 6 showed increased prevalence in AD (Fig. 6D, E). Spearman’s analysis showed a strong positive correlation (0.76) between the TOMM7 gene and activated CD8 T cells (*P* < .001) and a strong negative correlation (–0.59) with MDSC cells (*P* < .001; Fig. 6F).

### 3.7. Mapping of ceRNA networks for key genes and predictive potential of drugs

**Figure 7.**
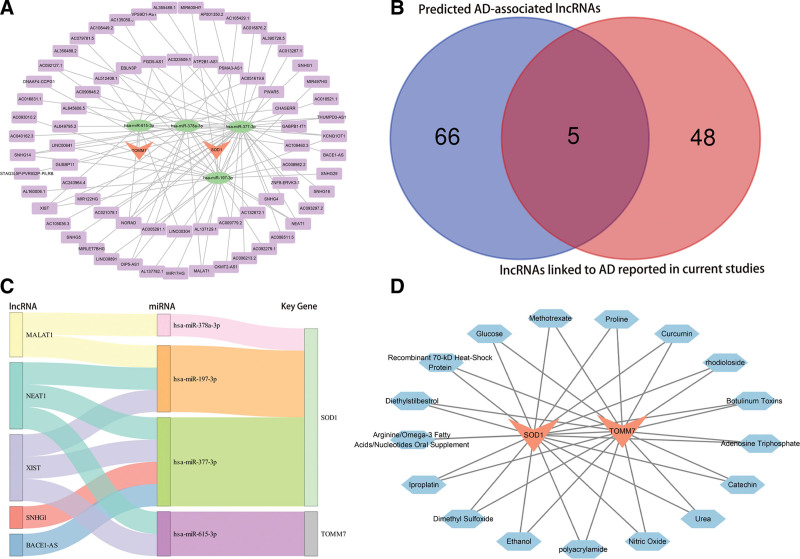
Combined analysis of the ceRNA regulatory network and drug prediction. (A) ceRNA network diagram (red triangles are mRNAs, green circles are miRNAs, lavender rectangles are lncRNAs). (B) The Venn diagram showing the overlap between AD-related lncRNAs and those predicted in this study has been documented in the literature. (C) Sankey diagram illustrating main LncRNA-miRNA-mRNA interactions. (D) Network map of key genes corresponding to therapeutic drugs *k* genes are shown in red triangles and corresponding compounds in blue hexagons). AD = Alzheimer’s disease.

**Figure 8.**
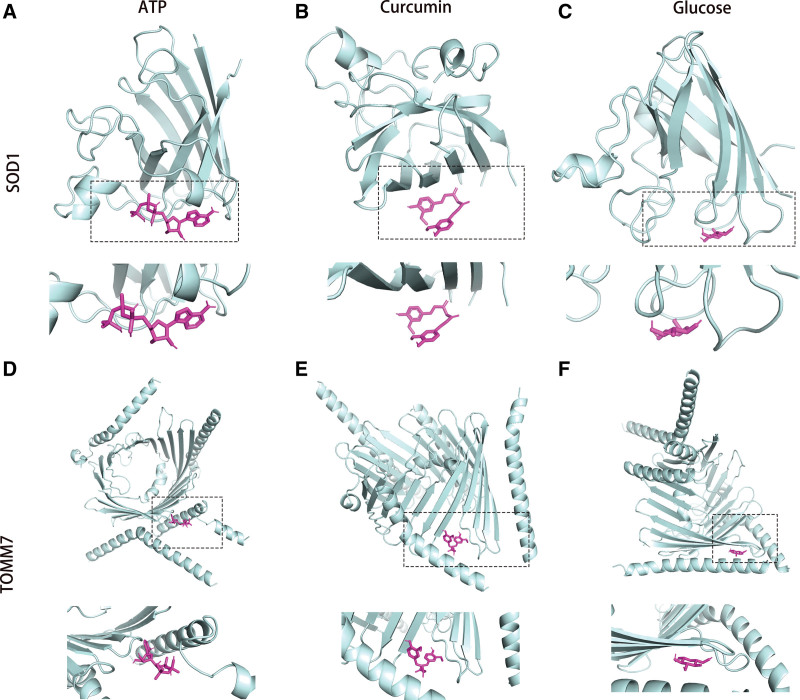
The molecular docking of curcumin with key genes. (A–F) Molecular docking revealed the binding of curcumin to its targets.

In the ceRNA prediction network mapping for key genes, we found that the 2 key genes interacted with 4 miRNAs (hsa-miR-615-3p, hsa-miR-378a-3p, hsa-miR-377-3p, hsa-miR-197-3p) and 71 lncRNAs (such as NORAD, NEAT1, CHASERR; Fig. 7A). By intersecting the lncRNAs related to AD reported in the current literature^[52-61]^ with the lncRNAs we predicted, we found that NEAT1, XIST, SNHG1, MALAT1 and BACE1-AS are the key lncRNAs related to mitochondrial dysfunction and PCD during the occurrence and development of AD (Fig. 7B). lncRNA-miRNA-mRNA network visualized by Sankey diagram (Fig. 7C). The coremine database predicted that the 2 key genes might correspond to 17 drugs (such as urea and nitric oxide;Fig. 7D). The binding energies between ligand and receptor were calculated by molecularly docking the protein structures of the key genes with the corresponding compounds. We observed favorable binding activities of SOD1 and TOMM7 with curcumin, with respective binding energies of −6.7 kcal/mol and −4.2 kcal/mol. (Fig. 8A–F; Table S1, Supplemental Digital Content, https://links.lww.com/MD/R561).

## 4. Discussion

Accumulating evidence indicates that mitochondria-associatedPCD pathways are pivotal in AD development and progression.^[24,65,66]^ Concurrently, studies have demonstrated that modulation of the mitochondrial apoptotic pathway – through mechanisms such as the oxidative stress reduction, stabilization of mitochondrial membrane potential, inhibition of cytochrome c translocation from mitochondria to the cytoplasm, and suppression of mitochondria-dependent, caspase-mediated apoptotic pathways, et al. – can significantly ameliorate AD symptoms.^[67-69]^ However, the mechanisms linking mitochondria-related PCD and AD have yet to be fully elucidated. Furthermore, current therapeutic approaches for AD do not adequately meet clinical needs. Consequently, this investigation primarily aims to investigate novel biomarkers related to mitochondrial function and PCD in the AD context, with the goal of creating innovative, effective, and safe treatment options for this condition.

This investigation detected 2 key genes, SOD1 and TOMM7, by means of 2 machine learning algorithms. SOD1, which is integral to the apoptotic signaling and the mitigation of oxidative stress within mitochondria,^[70-72]^ is a factor increasingly acknowledged as pivotal in the pathogenesis of AD.^[73,74]^ According to previous studies, SOD1 has been implicated in AD pathogenesis by influencing several pathways, such as mitochondrial dysfunction,^[73]^ cell apoptosis,^[75-77^, and deficits in memory and cognition of AD.^[78]^ In the AD context, dysregulation of SOD1 may lead to heightened oxidative impairment of mitochondrial components, culminating in mitochondrial dysfunction and subsequent PCD.^[76,77]^ This dysfunction is typified by a decrease in ATP generation, a decline in mitochondrial membrane potential, and the secretion of pro-apoptotic factors, including cytochrome c, which subsequently stimulate caspase pathways implicated in apoptosis.^[79,80]^ Besides, previous research suggested that SOD1 may interact with other mitochondrial proteins and pathways, thereby affecting mitochondrial dynamics and neuronal health.^[81]^ Specifically, the interaction between SOD1 and dynamin-related protein 1 is essential for preserving mitochondrial morphology and function, which is frequently compromised in AD.^[82,83]^ Furthermore, deleting the SOD1 gene leads to amyloid precursor protein (APP) oligomerization and memory loss in an AD mouse model, indicating that SOD1 may be participating in regulating APP metabolism in AD.^[84]^ Consequently, the impairment or altered expression or activity of SOD1 function may exacerbate mitochondrial dysfunction and contribute to neurodegeneration, highlighting its potential as a curative target in AD. The TOM complex is crucial for regulating the import of mitochondrial precursor proteins to preserve mitochondrial function under pathophysiological conditions.^[85]^ So far, 2 members of the complex, TOMM70 and TOMM40, are considered to be related to AD.^[85-87^. This study was the first to show that TOMM7 also serves as a pivotal factor in mitochondrial dysfunction and PCD in AD. TOMM7, one of the subunits of the TOMM complex, modulates its assembly and stability, which facilitates the efficient translocation of proteins from the cytoplasm to the mitochondria.^[88]^ Although there is no direct evidence that TOMM7 is involved in AD, another bioinformatics study showed that TOMM7 may be correlated with mild cognitive impairment (MCI) recently.^[89]^ Moreover, numerous investigations have illustrated that TOMM7 is essential for the maintenance of mitochondrial function.^[88,90,91]^ The deficiency of TOMM7 could disrupt the link between oxidation and ATP synthesis, altering mitochondrial function. As previously demonstrated, the underexpression of TOMM7 results in mitochondrial dysfunction and PCD, potentially participating in the progression and onset of AD. Comprehending the specific mechanisms by which TOMM7 influences mitochondrial health could offer insights into possible therapeutic targets for the mitigation of AD effects.

Regulation of mitochondrial function relies not only on intracellular autophagic mechanisms but also involves an emerging transcellular degradation process – transmitophagy.^[92,93]^ Centered on the internalization and degradation of neuron-derived damaged mitochondria by astrocytes, this process is pivotal for maintaining mitochondrial homeostasis in the brain.^[92]^ Lampinen et al first demonstrated in 5 × FAD mice and induced pluripotent stem cell (iPSC)-derived AD models that transmitophagy between neurons and astrocytes undergoes marked dysregulation, characterized by age-dependent excessive endocytosis and degradation of neuronal mitochondria by astrocytes. The underlying mechanisms involve aberrant formation of tunneling nanotubes (TNTs) and dysregulation of mitophagy-related factors (e.g., Ambra1), which ultimately impair the neuron-supporting functions of astrocytes and exacerbate neuronal damage.^[92]^ This finding expands the mitochondrial pathology of AD to a novel dimension of intercellular crosstalk. Notably, the key genes identified in this study, SOD1 and TOMM7, are both deeply integrated into the mitophagy regulatory network, suggesting they may serve as critical molecular hubs linking intracellular autophagic defects to aberrant transcellular transmitophagy. In the context of amyotrophic lateral sclerosis (ALS), mutant superoxide dismutase 1 directly impairs mitophagy by binding to and sequestering optineurin, a key receptor required for the formation of mitophagosomes. The sequestration of optineurin into mSOD1 aggregates reduces mitophagic flux, leading to the accumulation of dysfunctional mitochondria.^[94]^ Notably, in AD, SOD1 is also prone to aberrant posttranslational modifications and misfolding,^[95]^ which may confer upon it a similar, pathological gain-of-function ability to sequester optineurin and disrupt mitophagy. This may ultimately force neurons to clear these undigested damaged mitochondria via alternative pathways, such as enhanced transcellular transfer. Similarly, as a core subunit of the TOM complex, TOMM7 is essential for the stable accumulation of PINK1 on the outer mitochondrial membrane, thereby governing the canonical PINK1-Parkin autophagic pathway.^[96]^ Aberrant (downregulated) TOMM7 expression may directly compromise mitophagic capacity within neurons; this is consistent with our bioinformatic findings linking TOMM7 downregulation to mitochondrial dysfunction. Meanwhile, the integrity of the PINK1-Parkin pathway may also be required for astrocytes to efficiently recognize and process neuron-derived mitochondria. Thus, TOMM7 deficiency may disrupt transmitophagy balance through dual impairment of both the “supplier” (neurons) and the “processor” (astrocytes). Although the direct role of SOD1 and TOMM7 in transmitophagy remains unexplored, their established functions in mitochondrial import, redox balance, and mitophagy regulation position them as plausible mediators in this intercellular process. The dysregulation of SOD1 and TOMM7 in AD could potentially disrupt not only intracellular mitochondrial homeostasis but also the neuron-astrocyte crosstalk vital for clearing compromised mitochondria via transmitophagy. Therefore, investigating whether and how SOD1 and TOMM7 participate in transmitophagy could unravel a novel dimension of mitochondrial pathophysiology in AD and open new therapeutic avenues aimed at restoring intercellular mitochondrial quality control.

For additional comprehension of the role of these genes, we performed a GSEA enrichment analysis. Notably, these 2 genes showed similar trends of pathway enrichment. Both SOD1 and TOMM7 were functionally related to the chemokine signaling pathway, regulating the actin cytoskeleton, ribosome, and lysosome. Chemokine signaling pathways are well-established as having an essential function in neuroinflammatory processes and neuronal dysfunction in the context of AD.^[97-99]^ Specifically, chemokines and their receptors are implicated in several key aspects of AD pathology by activating downstream signaling pathways, such as, but not restricted to, Aβ deposition, tau protein hyperphosphorylation, and neuronal loss.^[97-99]^ Moreover, chemokines appear to exert multifaceted effects on brain function, including the control of neurogenesis and synaptic plasticity in regions associated with memory and cognitive capabilities.^[98]^ Researching how chemokines and their receptors function in AD could clarify the disorder’s mechanisms and support the development of innovative therapies. Regulation of the actin cytoskeleton is crucial in AD pathogenesis,^[100]^ as it maintains dendritic spine integrity essential for synaptic function.^[101]^ Disrupted actin dynamics can cause synaptic failure, contributing to AD-related cognitive deficits.^[100]^ Proteins like Cofilin-1 and CAP2, which regulate actin, are abnormally regulated in AD, leading to synaptic dysfunction.^[102,103]^ Understanding these mechanisms may reveal therapeutic targets for AD and other neurodegenerative disorders. Ribosomes are critical cellular structures involved in protein synthesis. While direct evidence linking ribosomes to AD is lacking, dysfunction in ribosomal activity is associated with reduced rates and capacities for protein synthesis. Such impairments in protein synthesis may denote one of the primary neurochemical changes detected in AD.^[104]^ Moreover, mitochondrial dysfunction is an AD well-documented feature,^[105]^ and ribosomes have a function in mitochondrial protein synthesis.^[106]^ Mitochondria rely on ribosomes for the synthesis of most of their proteins, which are crucial for maintaining mitochondrial function.^[107]^ Disruptions in ribosomal activity could, therefore, contribute to mitochondrial dysfunction, an AD hallmark. This connection between ribosomal function and mitochondrial health underlines the potential function of ribosomes in AD onset. Additionally, the function of ribosomal pathways in cellular survival and function has been explored in other contexts, such as chondrocytes, where an upregulated ribosome pathway was shown to improve cell survival and biofunction.^[108]^ This suggests that ribosomal pathways might similarly influence neuronal survival and function in the AD context, potentially offering novel pathways for therapeutic intervention. Lysosomes are gradually considered crucial players in AD pathogenesis.^[109,110]^ They act as both degradation centers and signaling hubs in cells, essential for cellular homeostasis, development, and aging.^[111]^ Previous studies showed that disruptions in lysosomal pathways in AD impair autophagy and mitophagy, resulting in damaged mitochondria buildup and oxidative stress, which exacerbate neuronal damage.^[112]^ Moreover, lysosomes interact with mitochondria to maintain cellular health, and disruptions in this interaction can contribute to AD.^[113]^ Besides, lysosomes have an essential function in iron metabolism and redox balance, and disturbances here can lead to neuronal death, implicating them in AD.^[114]^ Understanding the multifaceted function of lysosomes in AD may induce the development of innovative approaches to restore lysosomal function and alleviate disease progression.

In our study, the miRNA bound to SOD1 or TOMM7 was predicted. Then, we predicted the lncRNA corresponding to miRNA and constructed a lncRNA-miRNA-mRNA regulatory network. However, these miRNAs have been less studied to verify the connection to AD. Further clinical and basic studies are required to validate the functions of such miRNAs in AD development. In our study, we found that 5 lncRNAs, NEAT1, XIST, SNHG1, MALAT1, and BACE1-AS, may play a key role in mitochondrial dysfunction and PCD in AD by combining our predictions with the results of previous studies.^[52,54-61,115]^

NEAT1 has been proven to be elevated in AD brain tissue.^[116]^ Previous studies have demonstrated that the expression of NEAT1 is related to the characteristic aggregation of misfolded proteins, including amyloid-β and tau.^[116]^ Moreover, the downregulation of miR-27a-3p by NEAT1 exacerbated the pathological features of AD, including increased amyloid protein and tau phosphorylation, which are critical in the disease’s progression.^[117]^ Additionally, NEAT1 has been considered a potential blood-based biomarker for AD. Investigations illustrated that the NEAT1 plasma levels are substantially increased in AD individuals compared to healthy controls, indicating its utility in the early diagnosis and monitoring of the disorder.^[118]^ Regrettably, limited research exists on NEAT1’s impact on AD through mitochondrial function and PCD regulation; this needs to be substantiated by further research. XIST is involved in various biological processes^[119]^ and may significantly impact AD pathogenesis.^[120,121]^ One study indicated that XIST contributes to Aβ accumulation and neuroinflammation by repressing Aβ-degrading enzymes like neprilysin.^[120]^ In addition, reducing XIST in rat hippocampal neurons lessens Aβ-induced toxicity, oxidative stress, and apoptosis.^[121]^ XIST’s function in AD is further supported by its involvement in other neurological disorders, such as spinal cord injury, where its reduction aids recovery.^[121]^ These outcomes indicate that considering XIST as a target could be a promising treatment technique for AD. SNHG1 has a substantial role in several cellular processes,^[122]^ including regulating apoptosis and mitochondrial functions.^[123]^ For instance, in myocardial ischemia-reperfusion injury, SNHG1, modified by WTAP through m6A, plays a key role in regulating myocardial apoptosis, mitochondrial ROS generation, and polarization via the miR-361-5p/OPA1 pathway.^[123]^ Thus, we hypothesized that SNHG1 may affect AD by modifying mitochondrial function and PCD, indicating it as a possible therapeutic target for slowing AD progression. MALAT1 has emerged as a substantial factor in AD,^[124,125]^ but its role remains controversial. While some studies indicate that MALAT1 suppresses neuron apoptosis and inflammation while promoting neurite outgrowth,^[125,126]^ other research shows that reducing MALAT1 can counteract high glucose effects on mTOR activity and p-tau levels, potentially mitigating dementia.^[127]^ In cardiomyopathy, lower MALAT1 levels enhance mitochondrial autophagy and improve cell viability.^[128,129]^ Therefore, we hypothesize that MALAT1 may worsen AD by disrupting mitochondrial function, necessitating further investigation into its role. BACE1-AS, which positively regulates the expression of BACE1. Markers of oxidative stress and Aβ burden are related to BACE1 activity in AD patients.^[130,131]^ In the brains of AD individuals and mouse models, BACE1 accumulation was in both normal and dystrophic presynaptic terminals that surround amyloid plaques, leading to an elevation in Aβ synthesis near synapses.^[132]^ Furthermore, the inhibition of BACE1-AS mitigated neuronal damage by modulating autophagy via the miR-214-3p/ATG5 signaling pathway in AD.^[133]^ Meanwhile, although multiple BACE1 suppressors have demonstrated therapeutic effects in AD animal models, numerous others have been deemed unsuitable for further human development owing to safety concerns.^[134,135]^ Thus, BACE1-AS may act as a prospective diagnostic biomarker and treatment target for AD.

To explore the link between key genes and immunity, we analyzed immune infiltration and discovered that activated CD8 T cells and MDSC are strictly related to key genes. Recent investigations have illustrated that CD8+ T cells infiltrate the brains of AD individuals and exhibit altered mitochondrial dynamics, which may contribute to neuroinflammation and neuronal dysfunction.^[136]^ Specifically, CD8+ T cells in the AD brain regulated the neuronal and synapse-related gene expression, indicating their potential role in modulating synaptic plasticity and cognitive decline.^[136]^ Moreover, mitochondrial dysfunction in CD8+ T cells has been linked to their impaired effector functions, which is particularly evident in chronic conditions, including hepatitis B virus infection.^[137]^ Additionally, the metabolic characteristics of CD8+ T cell subsets, including their reliance on mitochondrial respiration, are crucial for their activation and differentiation.^[138]^ In AD, the presence of CD8+ T cells with mitochondrial dysfunction and senescence characteristics has been noted, suggesting that these cells may participate in the disorder’s pathophysiology.^[139]^ The next immune cells that are associated with key genes to be addressed are the MDSCs. In early-stage amnestic mild cognitive impairment linked to AD, circulating monocytes and granulocyte MDSCs double in number. This increase is due to heightened expression of inflammatory mediators and hypoxia regulation in AD, which promote MDSC expansion and activation, leading to immunosuppression in inflammatory tissues.^[140-142]^ Various chemokines and growth factors secreted by MDSCs, including interleukin-10 and transforming growth factor-beta, serve as potent inducers and maintenance factors that facilitate the differentiation of microglia into the M2anti-inflammatory phenotype. This differentiation supports the maintenance of an anti-inflammatory environment and promotes tissue repair processes. Nevertheless, MDSCs present a dualistic nature, as they can exert detrimental effects when tissue damage persists, and inflammation transitions into a chronic phase.^[141]^ Previous studies demonstrated that MDSCs accumulate in tissues and negatively impact chronic conditions like tumors and age-associated disorders by hindering the removal of senescent and cancer cells and disrupting energy metabolism and tissue proteostasis.^[143]^ In addition, MDSCs deplete arginine and tryptophan in inflammatory tissues by secreting arginase 1 and indoleamine 2,3-dioxygenase 1, hindering inflammatory cell growth, potentially causing neuronal atrophy,^[141]^ which could worsen AD symptoms.^[144]^ The precise mechanism by which MDSCs influence AD remains unclear and warrants further investigation through additional studies. In summary, all of the above underscore the importance of exploring immune cell metabolism as a potential avenue for therapeutic strategies in AD.

In the present, numerous treatments are available for AD, but all have unsatisfactory curative effects.^[145]^ By analyzing hub genes, we were able to identify 17 candidate pharmacological compounds that could aid in developing innovative AD treatments. Notably, ATP, glucose, and curcumin demonstrated the strongest correlations with the genes SOD1 and TOMM7. Consequently, these 3 predicted compounds could be candidate drugs for AD. As is well known, abnormal glucose metabolism significantly contributes to AD by triggering various pathogenic factors, including oxidative stress, mitochondrial dysfunction, and apoptosis.^[146]^ Meanwhile, glucose uptake is the first and rate-limiting step of glucose metabolism,^[147]^ and ATP is the principal end-product caused by glucose breakdown throughout glycolysis.^[148]^ Then glucose metabolism and ATP have been regarded as an efficient therapy for treating AD. For example, earlier research indicated that performance deficits observed in aging individuals during cognitive task training are attributable to inadequate cerebral glucose supply. Enhancing glucose availability in specific brain regions has been shown to influence cognitive performance, particularly in aged subjects, positively. Microinjections of glucose into the medial septum, hippocampus, striatum, and amygdala have been demonstrated to improve memory processing.^[149-151^ In addition, ATP synthase, a proton pump that converts the chemical potential energy from the proton gradient across the inner mitochondrial membrane, created by the electron transport chain, into ATP, has been shown to be effective in the treatment of both cell and animal models of AD.^[152]^ However, it will take a long time for these initial encouraging results to be validated. Curcumin, another candidate drug, is a primary bioactive polyphenol extracted from *Curcuma longa* rhizomes of the *Zingiberaceae* family, which has significant biochemical and biological effects, including anti-inflammatory, antiviral, antibacterial, antioxidant, and anticancer properties.^[153-155]^ Many studies on AD show that curcumin protects SH-SY5Y neuroblastoma cells from Aβ-induced mitochondrial damage, inhibits apoptosis, restores antioxidant enzyme function, reduces oxidative stress, and ultimately protects neurons.^[156-159]^ Additionally, curcumin targets various therapeutic biochemical and molecular pathways for AD, such as transcription factors, inflammatory cytokines, and anti-apoptosis proteins.^[160,161]^ However, the limited bioavailability and selectivity of curcumin significantly constrain its therapeutic application. Consequently, enhancing its pharmacokinetic profile and designing curcumin analogs represent promising strategies for the treatment of AD.

In this study, 2 pivotal genes linked to mitochondrial function and PCD in AD were identified through bioinformatics analysis utilizing the GEO database. Subsequent analyses were conducted based on these key genes, including GSEA, immune infiltration assessment, molecular regulatory network construction, and drug prediction. The outcomes offer important insights into the pathogenesis, diagnosis, and therapeutic strategies for AD. However, our investigation has several limitations. Initially, the information from several web sources may introduce variability and bias. Second, while we found a link between SOD1, TOMM7, mitochondrial function, and PCD in AD, there is limited literature on these DEGs and AD, requiring additional validation through in vitro and in vivo trials. Third, our analysis is based on blood samples from AD individuals and controls and needs additional validation in specific brain regions like the frontal cortex and hippocampus. These limitations highlight areas for further investigation and opportunities for other teams to participate.

## 5. Conclusion

The genes SOD1 and TOMM7, which are associated with mitochondrial function and PCD, exhibited significant differential expression between AD patients and normal controls. This finding underscores the critical function of mitochondrial and PCD mechanisms in the pathogenesis of AD and suggests that these DEGs may act as possible targets for the diagnosis and therapeutic intervention of the disease.

## Acknowledgments

The authors confirm that all individuals listed in the Acknowledgments have given written permission for their names to be included.

## Author contributions

**Conceptualization:** Hua-xiong Zhang, Hong-yan Li.

**Data curation:** Hua-xiong Zhang.

**Formal analysis:** Hua-xiong Zhang.

**Funding acquisition:** Hua-xiong Zhang.

**Investigation:** Hua-xiong Zhang.

**Methodology:** Hua-xiong Zhang, Hong-yan Li.

**Resources:** Hua-xiong Zhang.

**Software:** Hua-xiong Zhang.

**Supervision:** Hua-xiong Zhang.

**Validation:** Hua-xiong Zhang.

**Visualization:** Hua-xiong Zhang.

**Project administration:** Hong-yan Li.

**Writing – original draft:** Hua-xiong Zhang.

**Writing – review & editing:** Hong-yan Li.

## Supplementary Material


