# Supplementary material for: Mitochondrial dysfunction and programmed cell death in Alzheimer’s disease: A retrospective bioinformatics study
Source: Medicine (Baltimore). 2026 Mar 20;105(12):e48105. doi: 10.1097/MD.0000000000048105 (PMC13008200; doi:10.1097/MD.0000000000048105)
Supplement: Supplementary file 1 [file medi-105-e48105-s001.docx]

Table S1. Results of molecular docking

| Symbol | PDB | Molecule Name | PubChem CID | affinity(kcal/mol) | hydrogen bonds |
| --- | --- | --- | --- | --- | --- |
| SOD1 | 1MFM | Adenosine Triphosphate | 5957 | -3.13 | 1 |
|  |  | Curcumin | 969516 | -6.7 | 2 |
|  |  | Glucose | 5793 | -2.08 | 4 |
| TOMM7 | 7CK6 | Adenosine Triphosphate | 5957 | -1.71 | 1 |
|  |  | Curcumin | 969516 | -4.2 | 0 |
|  |  | Glucose | 5793 | -1.56 | 2 |

Abbreviations: SOD1£ºsuperoxide dismutase 1£»TOMM7 £ºtranslocase of the outer mitochondrial membrane 7
